# The use of Guedel Airway for Naso-Orogastric tube insertion in patients with COVID-19. A way to minimize aerosol generation

**DOI:** 10.12669/pjms.36.COVID19-S4.2765

**Published:** 2020-05

**Authors:** Faraz Shafiq, Dileep Kumar

**Affiliations:** 1 Dr. Faraz Shafiq, Assistant Professor, Department of Anaesthesiology, The Aga Khan University, Karachi, Pakistan; 2 Dr. Dileep Kumar, Assistant Professor, Department of Anaesthesiology, The Aga Khan University, Karachi, Pakistan

Dear Editor,

The Oro/Naso gastric tube insertion is an important aspect of management in patients diagnosed to have COVID 19. The procedure should be considered as high risks, with potential to generate aerosols. The recent recommendations by Royal College of Anesthetists England, “**AIDE-MEMOIRE:** Nasogastric tube placement checks before first use in critical care settings during the COVID-19 response”, reinforces the need of Chest radiograph to confirm its placement. The publication also discouraged the practice of commonly doing “*Whoosh test”* for this purpose. As, this would be risky in terms of generating aerosols. However, one should not forget the importance of successful placement of gastric tube using single attempt. The overall failure rate is around 50-60%.^1^ For various reasons, this failure is expected to be increased during this pandemic. Besides, repeated attempts can lead to increased risk of exposure and getting infected. Many techniques have been reported in literature to overcome this failure and to increase the chance of first pass success. In our opinion, use of adequate size *Guedel airway* is best strategy to lift the tongue and decrease the resistance down the oropharynx, hence increase the chances of passing tube in first attempt.^2^ The already reported technique would be really useful technique for decreasing procedural time, increasing the first pass success and hence minimizing the aerosol generation in current scenario.

## References

[1] Appukutty J, Shroff PP (2009). Nasogastric tube insertion using different techniques in anesthetized patients:a prospective, randomized study. Anesth Analg.

[2] Shafiq F, Hameed F, Siddiqui K (2018). Use of Guedel Airway as a guide to insert nasogastric tube under general anaesthesia:A simple and logical way. Pak J Med Sci.

